# Co-occurrence of moyamoya syndrome and Turner syndrome in a 4-year-old Sudanese girl: a rare case report

**DOI:** 10.1186/s13256-026-06193-7

**Published:** 2026-06-04

**Authors:** Rayan Khalid, Imad Fadl-Elmula

**Affiliations:** 1 Department of Clinical Genetics and Immunology, Assafa College, Khartoum, Sudan; 2 Department of Clinical Genetics, Al Neelain Stem Cell Center, Al Neelain University, Khartoum, Sudan; 3 Elite Center for Genetic Services, Khartoum, Sudan

**Keywords:** Turner syndrome, Moyamoya syndrome, Recurrent stroke, Chromosomal analysis, Sudan

## Abstract

**Background:**

Moyamoya syndrome (MMS) is a chronic cerebrovascular condition characterized by progressive stenosis of the internal carotid arteries and the development of fragile collateral vessels, producing the characteristic “puff of smoke” appearance on angiography. This syndrome is uncommon in African populations, with higher prevalence in East Asia, with reported incidence rates ranging from 0.43 to 2.3 per 100,000 populations. Its co-occurrence with Turner syndrome (TS) is exceptionally rare, with only a handful of cases reported globally. This report describes the clinical features, diagnostic challenges, and management constraints of TS-associated MMS in a 4-year-old Sudanese girl.

**Case presentation:**

A 4-year-old Sudanese girl was referred to the Elite Center for Genetic Services due to dysmorphic features consistent with TS and a history of recurrent transient ischemic attacks and right-sided hemiparesis. Clinical examination revealed an asymmetrical triangular face, depressed nasal bridge, epicanthal folds, low posterior hairline, webbed neck, wide elbow angles, and widely spaced nipples. Neurological assessment confirmed right-sided motor weakness of grade 3/5 in upper and lower limbs, with intact sensation. Hemoglobin electrophoresis excluded sickle cell disease. Brain MRI showed acute-on-chronic left hemispheric infarction. Magnetic resonance angiography (MRA) demonstrated bilateral internal carotid artery stenosis and characteristic collateral vessels, findings suggestive of moyamoya syndrome. Karyotyping confirmed monosomy X (45,X), establishing the diagnosis of Turner syndrome. Due to limited access to revascularization surgery in Sudan, conservative management was initiated with aspirin (5 mg/kg/day), physical therapy, hormonal evaluation, and multidisciplinary follow-up.

**Conclusion:**

This case highlights the diagnostic and therapeutic challenges of managing rare neurogenetic syndromes in resource-limited, conflict-affected settings. Early recognition of the TS–MMS association, and differentiation from more common causes like sickle cell disease, is critical to reducing stroke recurrence and long-term disability in vulnerable pediatric populations.

## Background

Turner syndrome (TS) is a rare genetic disorder caused by complete or partial monosomy of the X chromosome, occurring in approximately 1 in 2,500 live female births [1]. It is classically characterized by short stature, gonadal dysgenesis, congenital cardiac anomalies, and distinctive dysmorphic features, such as a webbed neck, low posterior hairline, and widely spaced nipples [2, 3]. While the phenotypic spectrum and systemic vascular complications of TS, including aortic dilation and coarctation, are well documented, its association with progressive cerebral arteriopathy remains uncommon [4].

Moyamoya syndrome (MMS) is a rare form of chronic, secondary intracranial vasculopathy characterized by progressive stenosis of the terminal internal carotid arteries and the compensatory development of fragile collateral networks that produce a “puff of smoke” appearance on angiography [5]. Unlike idiopathic moyamoya disease, MMS occurs in the context of an underlying condition, such as sickle cell disease, autoimmune disorders, or certain genetic syndromes, and requires identification of this primary etiology for appropriate management [6]. This condition exhibits a bimodal age distribution, with a pediatric peak between 5 and 10 years, often presenting with ischemic symptoms, and a second peak in adulthood (30–50 years) typically associated with hemorrhage [7]. Globally, MMS incidence is highest in East Asia, while data from sub-Saharan Africa remain extremely limited, with most reported cases linked to sickle cell disease [8].

The co-occurrence of TS and MMS has been reported only sporadically in the literature. Although a few cases have been described globally, to date, no genetically confirmed cases have been reported from Africa [9]. In regions where hemoglobinopathies predominate as causes of pediatric stroke, non-hemoglobinopathic etiologies may be underrecognized, particularly when access to advanced neuroimaging and modern genetic investigations is limited. This diagnostic gap can delay interventions that reduce the risk of recurrent cerebrovascular events [10].

This case report describes a 4-year-old Sudanese girl who presented with classic features of Turner syndrome and neuroimaging findings consistent with moyamoya syndrome.

## Case presentation

A 4-year-old Sudanese girl was referred to the Elite Center for Genetic Services in Khartoum for evaluation of short stature and dysmorphic features evident since early childhood. She was the only child of non-consanguineous parents (father aged 47, mother 34 years), and she was born at term via unassisted vaginal delivery at home, with no perinatal complications. Her mother reported global growth delay from birth.

Six months prior to referral, she experienced a transient syncopal episode followed by brief right-sided limb weakness. One month before genetic evaluation, she was admitted to a pediatric tertiary hospital following a generalized tonic–clonic seizure and loss of consciousness, which resulted in persistent right hemiparesis. Since then, she has been on aspirin 5 mg/kg/day, has received regular physical therapy, three sessions per week, and has remained seizure-free without antiepileptic drugs. She had no history of recurrent infections, vaso-occlusive crises, or family history of miscarriages, dysmorphic features, or neurodevelopmental disorders.

**Fig. 1:**
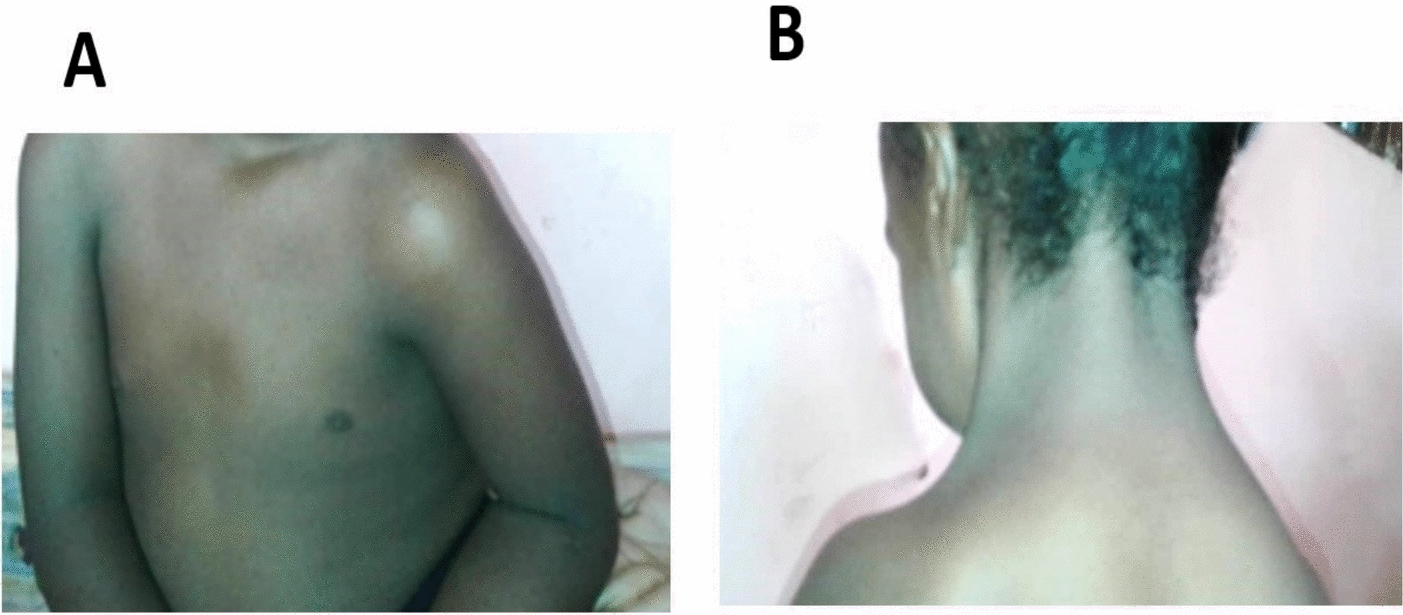
Features of a patient with Turner syndrome and moyamoya syndrome: **A** Anterior trunk view demonstrating widely spaced nipples and right upper limb posturing consistent with hemiparesis. **B** Posterior view of the neck and upper back showing classic webbed neck in Turner syndrome

On examination, she was afebrile, hemodynamically stable, and alert. Anthropometry revealed weight 12.6 kg (< 10th percentile) and height 92 cm (< 3rd percentile), consistent with proportionate short stature. Dysmorphic features included an asymmetrical triangular face, depressed nasal bridge, epicanthal folds, low posterior hairline, webbed neck, and widely spaced nipples (Fig. 1). Neurological examination confirmed right-sided motor weakness (grade 3/5 in upper and lower limbs) with intact sensation.

**Fig. 2:**
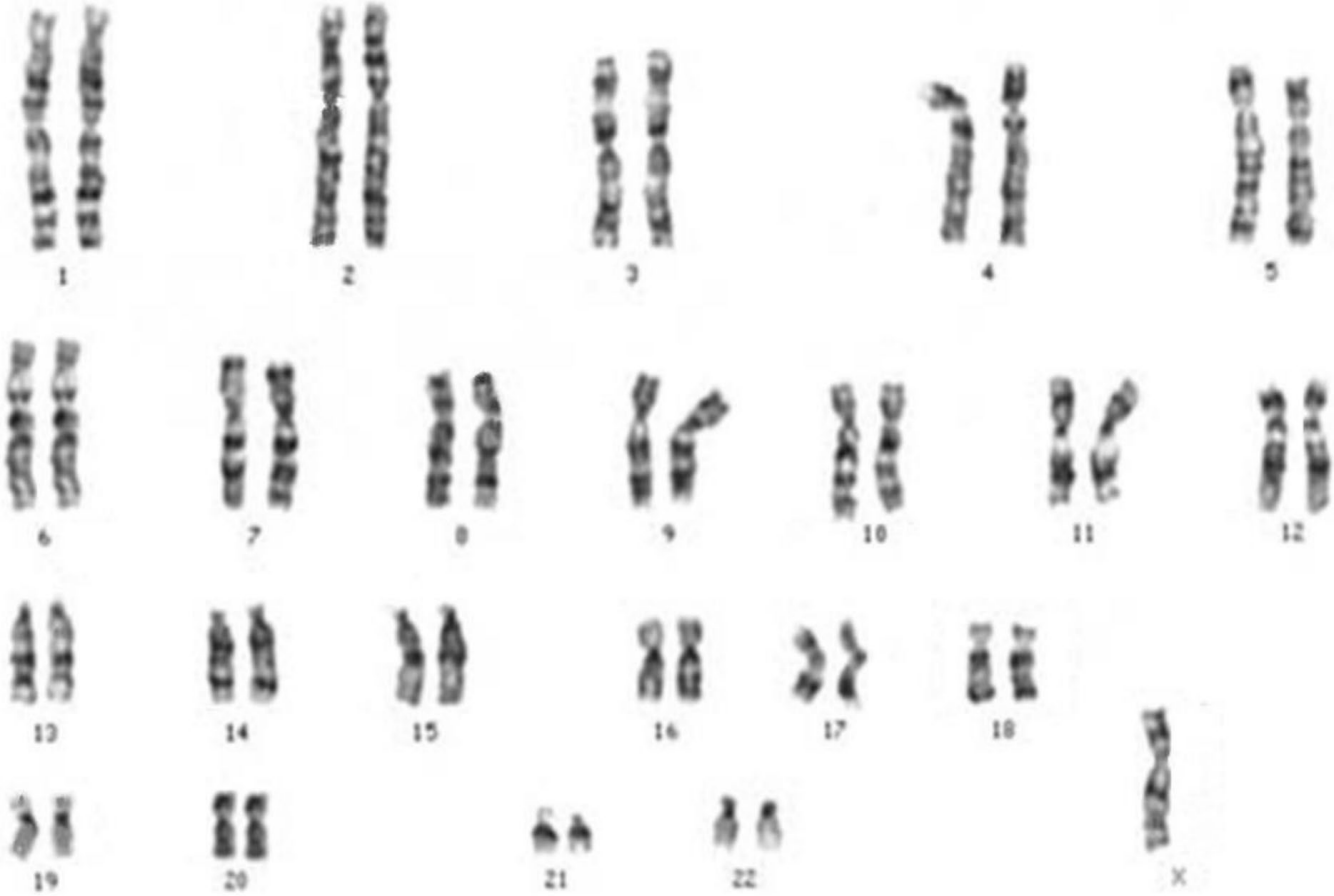
Chromosomal analysis for the patient showed 45,X karyotype consistent with clinical diagnosis of Turner’s syndrome

Laboratory investigations, including complete blood count, erythrocyte sedimentation rate, renal function, lipid profile, prothrombin time, sickling test, and hemoglobin electrophoresis (showing normal HbA), were unremarkable, excluding sickle cell disease (SCD). Cardiac imaging (echocardiography, ECG, chest X-ray) and abdominal ultrasound were normal. Brain MRI demonstrated acute-on-chronic left hemispheric infarction with mild cerebral atrophy. Magnetic resonance angiography (MRA) revealed bilateral internal carotid artery stenosis and characteristic “puff of smoke” collateral vessels, findings suggestive of moyamoya syndrome. Karyotyping confirmed monosomy X (45,X), establishing the diagnosis of Turner syndrome (Fig. 2).

Comprehensive genetic counseling was provided to the parents, addressing the implications of Turner syndrome, the increased risk of cerebrovascular events due to coexisting moyamoya syndrome, and the importance of long-term surveillance. Due to limited access to revascularization surgery in Sudan, management remained conservative. Growth hormone therapy was deferred pending formal endocrine evaluation. At 3-month follow-up, the patient showed improved motor strength (grade 4/5), no seizure recurrence, and continued adherence to aspirin and physiotherapy. A multidisciplinary care plan involving clinical genetics, endocrinology, neurology, cardiology, and rehabilitation services was established to optimize long-term outcomes.

## Discussion

The co-occurrence of TS and MMS remains exceptionally rare globally [9, 11]. While the precise pathophysiological link is incompletely understood, emerging evidence supports a shared vasculopathic mechanism rooted in X-chromosome haploinsufficiency. Genes located on the short arm of the X chromosome, particularly tissue inhibitor of metalloproteinases-1 (*TIMP1*) and filamin A (*FLNA*), are critical for vascular wall integrity and endothelial function [12, 13]. Their deficiency may lead to unopposed matrix metalloproteinase activity, smooth muscle hyperplasia, and progressive stenosis of intracranial arteries, features also observed in systemic vasculopathies associated with TS, such as aortic dilation and coarctation [12]. Additionally, dysregulation of transforming growth factor-beta (TGF-β) signaling, previously implicated in both Marfan syndrome and TS-related aortopathy, may further contribute to intimal thickening and aberrant collateral vessel formation characteristic of MMS [14].

Importantly, MMS has also been reported in association with other genetic conditions, including Down syndrome, neurofibromatosis type 1, and Klinefelter syndrome, suggesting a broader spectrum of genetically mediated cerebrovascular fragility [6]. This reinforces the need for comprehensive evaluation of underlying syndromes in children presenting with unexplained stroke, particularly when dysmorphic features are present.

Sudan has one of the highest burdens of SCD worldwide, partly attributed to endemic malaria and high consanguinity marriage rate, which increases the burden of cerebrovascular complications such as MMS. Although TS has been identified as a genetic contributor to MMS, in Sudan and many other African countries, SCD is the most prevalent cause of MMS [15]. Previous studies showed that the diagnostic landscape in Sudan is dominated by SCD which accounts for up to 15% of pediatric MMS cases due to chronic hemolysis, recurrent vaso-occlusion, and mechanical endothelial injury [15]. Unlike TS-associated MMS, SCD-related vasculopathy typically occurs in the absence of recognizable dysmorphic phenotypic markers [15, 16].

In our case, the patient's constellation of classic TS features, including a webbed neck, widely spaced nipples, an asymmetrical triangular face, and proportionate short stature, served as highly visible physical indicators that should ideally trigger genetic evaluation early in the clinical course. In such cases, early genetic confirmation of TS enabled coordinated endocrine and cardiac surveillance, which may mitigate long-term complications and growth failure [17]. Notably, in the present case, recurrent cerebrovascular events occurred within 6 months of initial presentation, a pattern consistent with reported stroke recurrence rates of up to 30% within five years among untreated moyamoya syndrome patients [16]. While definitive revascularization surgery remains the standard of care in high-resource settings [18], its inaccessibility necessitated a conservative approach centered on antiplatelet therapy, rehabilitation, and close clinical follow-up.

This case report has distinct diagnostic limitations that reflect the realities of practicing in a resource-constrained setting. The patient was evaluated based on MRA alone because conventional catheter-based digital subtraction angiography (DSA), the current gold standard, was unavailable. Therefore, definitive angiographic staging could not be performed. While MRA is highly sensitive, it can overestimate luminal stenosis or miss subtle collateral patterns [19]. Furthermore, whole-exome sequencing was inaccessible to evaluate for modifying variants in *RNF213*, *ACTA2*, or other genes linked to primary or secondary moyamoya phenotypes [20]. Despite these diagnostic constraints, to our knowledge, this report represents the first genetically confirmed case of TS-MMS co-occurrence in sub-Saharan Africa.

## Conclusion

Early recognition of dysmorphic features in children presenting with stroke in high sickle cell disease prevalence regions may prompt timely evaluation for underlying genetic conditions such as Turner syndrome. When advanced neuroimaging or surgical revascularization is scarce, conservative management, including antiplatelet therapy, rehabilitation, and multidisciplinary follow-up, can help reduce the risk of recurrent events. This case illustrates how integrating basic genetic assessment into clinical evaluation may improve diagnostic accuracy in resource-limited settings.

## Data Availability

No datasets were generated or analyzed during the current study.

## Abbreviations

ACA: Anterior cerebral artery
DSA: Digital subtraction angiography
ICA: Internal carotid artery
MCA: Middle cerebral artery
MMS: Moyamoya syndrome
PCA: Posterior cerebral artery
PSS: Proportionate short stature
SCD: Sickle cell disease
TS: Turner syndrome
MRA: Magnetic resonance angiography
